# Sternal Closure After Clamshell Thoracotomy for Bilateral Lung Transplantation: Comparison Between Different Techniques

**DOI:** 10.1093/icvts/ivag079

**Published:** 2026-03-18

**Authors:** Marco Mammana, Giulia Pagliarini, Chiara Anna Schiena, Gabriella Roca, Viola Sambataro, Monica Loy, Marco Schiavon, Andrea Dell’Amore

**Affiliations:** Thoracic Surgery Unit, Department of Cardiac, Thoracic, Vascular Sciences and Public Health, University of Padua, Padua 35128, Italy; Thoracic Surgery Unit, Department of Cardiac, Thoracic, Vascular Sciences and Public Health, University of Padua, Padua 35128, Italy; Thoracic Surgery Unit, Department of Cardiac, Thoracic, Vascular Sciences and Public Health, University of Padua, Padua 35128, Italy; Thoracic Surgery Unit, Department of Cardiac, Thoracic, Vascular Sciences and Public Health, University of Padua, Padua 35128, Italy; Thoracic Surgery Unit, Department of Cardiac, Thoracic, Vascular Sciences and Public Health, University of Padua, Padua 35128, Italy; Thoracic Surgery Unit, Department of Cardiac, Thoracic, Vascular Sciences and Public Health, University of Padua, Padua 35128, Italy; Thoracic Surgery Unit, Department of Cardiac, Thoracic, Vascular Sciences and Public Health, University of Padua, Padua 35128, Italy; Thoracic Surgery Unit, Department of Cardiac, Thoracic, Vascular Sciences and Public Health, University of Padua, Padua 35128, Italy

**Keywords:** clamshell, bilateral lung transplantation, sternal closure

## Abstract

**Objectives:**

Clamshell thoracotomy is a common approach for bilateral lung transplantation; however, after chest closure, the 2 sternal ends may not reapproximate properly. We aimed to assess the efficacy of different techniques of sternal closure in determining a correct sternal alignment postoperatively.

**Methods:**

We performed a single-centre retrospective review of all patients who underwent bilateral lung transplantation through clamshell thoracotomy from 2016 to 2023. Patients were divided according to the sternal closure technique into the figure-of-8, resorbable suture (FRS), interrupted wired sutures (IWSs), and crossed wired sutures (CWSs) groups. Sternal alignment was evaluated on lateral chest X-rays and scored as normal, override, or separation.

**Results:**

Of the 164 eligible patients, the FRS, IWS, and CWS groups consisted of 44, 10, and 110 patients, respectively. Sternal separation was observed in 31 patients (18.9%), and its rate was significantly lower in the CWS group (9.1% compared to 40.9% and 30.0% in the FRS and IRS groups, respectively, *P* < .001). At multivariable analysis, increasing body mass index was associated with higher risk of sternal separation (odds ratio [OR]: 1.13, 95% confidence interval [CI]: 1.02-1.27), while the CWS technique was associated with a reduced risk (OR: 0.14, 95% CI: 0.06-0.34). Patients with sternal separation had significantly higher pain scores on postoperative day 7 and a trend towards significantly higher pain scores on postoperative day 14 (*P* = .056).

**Conclusions:**

In our study, the CWS technique led to better sternal alignment than other techniques and should be the preferred method of sternal closure after clamshell thoracotomy. Severe sternal misalignment is associated with higher pain scores early after transplantation.

## Graphical abstract

**Figure:**
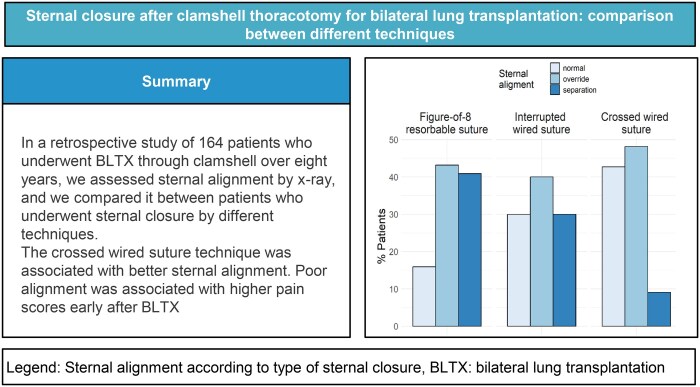

## INTRODUCTION

Lung transplantation represents the last therapeutic option for patients with end-stage pulmonary disease, offering significant improvements in both survival and quality of life.^1^

Bilateral lung transplantation (BLTX) is often performed through a clamshell incision, which provides an excellent exposure of the hilar structures and facilitates central cannulation for extracorporeal membrane oxygenation (ECMO).^2^ However, compared to bilateral anterior thoracotomy without sternal division, the clamshell incision may be associated with complications such as osteomyelitis, sternal override, and pain.^3^^,^^4^ Therefore, a good reapproximation of the 2 sternal ends at the time of chest closure is of particular concern. In fact, misalignment of the sternal ends may lead to sternal instability or pain.^5^

Several techniques have been proposed to achieve a firm reapproximation of the sternum after clamshell thoracotomy, including rigid plates,^6^^,^^7^ pins,^8^ or crossed wired sutures.^9^

However, there are only few, small comparative studies between different reapproximation techniques.^6^^,^^9^ These studies often relate the adequacy of sternal closure to the onset of postoperative complications (sternal dehiscence or infection). However, the impact of a poor sternal alignment on postoperative pain is unknown.

The aim of this study is to compare the efficacy of 3 different techniques of sternal closure on the alignment of the sternal ends, as evaluated by lateral chest X-rays (CXR), in patients undergoing clamshell thoracotomy for BLTX, and to relate the alignment of the sternal ends to postoperative pain.

## METHODS

### Ethical statement

This study was approved by the Institutional Review Board of the Padua University Hospital (approval number: 467n/AO/24). The study was conducted using anonymized data derived from an institutional database established and managed in accordance with the World Medical Association (WMA) Declaration of Taipei. The database and its ongoing use were approved and are monitored by the local research ethics committee. The requirement for individual informed consent was waived due to the anonymized and aggregated nature of the data.

### Patients and variables

We retrospectively reviewed of all patients who underwent BLTX at the Thoracic Surgery Unit of Padua University Hospital (Padua, Italy) between January 2016 and December 2023. Predefined exclusion criteria were: (1) single lung transplantation, (2) other surgical approach than clamshell incision (eg, double anterior thoracotomy), (3) did not survive to have at least one postoperative lateral CXR (in the upright position), (4) incomplete information about any of the collected variables.

During the study period, at our centre, the surgical approach to BLTX changed as a result of a concurrent modification in our policy regarding the use of intraoperative venoarterial extracorporeal membrane oxygenation (VA-ECMO). In the early period, clamshell incision was reserved for more difficult cases or when it was necessary to resort to intraoperative extracorporeal life support (selective VA-ECMO strategy). Starting from 2020, based on the positive experience reported by other centres,^10^ we progressively switched to a routine VA-ECMO strategy for all patients. This approach provides improved haemodynamic stability, protective lung ventilation, prolonged controlled reperfusion, and minimizes right heart strain.^11^^,^^12^ Consequently, most BLTX procedures have since been performed using a clamshell approach to facilitate central cannulation.

**Figure 1.**
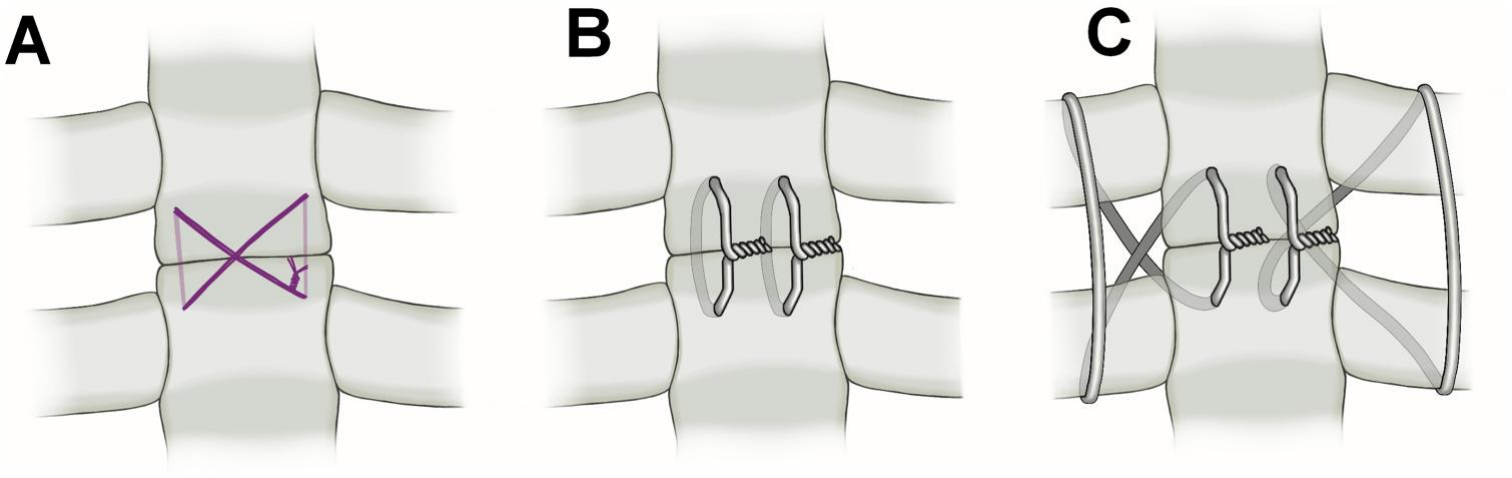
Different techniques for sternal fixation during closure of the clamshell thoracotomy. Figure-of-8, resorbable suture with a polydioxanone, size 1, loop suture (A); 2 interrupted sutures with steel wires (B); 2 crossed wired sutures with steel wires (C).

At the time of chest closure, the 2 sternal edges were reapproximated and fixed, according to the surgeon’s preference, either by a figure-of-8 stitch with a polydioxanone, size 1, loop suture (**Figure 1A**); by 2 interrupted sutures with steel wires (**Figure 1B**); or by 2 crossed wired sutures with steel wires (**Figure 1C**). The technique for the crossed wired suture is similar to the parasternally crossed suture described by Koster *et al*,^9^ with the difference that the wire ends lie over the body of the sternum, rather than over the intercostal space (**Figure 1C**).

During the study period, patients were cared for by a stable multidisciplinary team both in the ICU and in the surgical ward, with no major changes in staff composition, following unchanged protocols.^13^ In particular, the immunosuppressive regimen consisted of the association of calcineurin inhibitors, cell cycle inhibitors, and corticosteroids at a standard dose,^12^ while pain management consisted of the association of epidural anaesthesia with scheduled intravenous (or oral) agents (paracetamol and NSAIDs). Opioids (tramadol or morphine) were administered as needed in cases of uncontrolled pain.^14^

As soon as patients were transferred from the ICU to the ward, the nursing staff routinely recorded patient-reported pain scores according to the numeric rating scale (NRS) 3 times a day. Anteroposterior and laterolateral CXR were performed according to the clinical need, without a fixed schedule, but at least on a weekly basis. Patients with a normal postoperative course underwent a scheduled bronchoscopy with transbronchial biopsies at 1 month from transplantation, after which, in the absence of rejection, they were discharged. The first follow-up visit was scheduled 1 month after discharge (about 60 days from transplantation).

Baseline demographics, preoperative clinical data, intraoperative details, and early postoperative outcomes were collected for each patient. Patients were stratified into 3 groups based on the method of sternal closure: figure-of-8, resorbable sutures (FRSs), interrupted wired sutures (IWSs), and crossed wired sutures (CWSs) (**Figure 1**).

**Figure 2.**
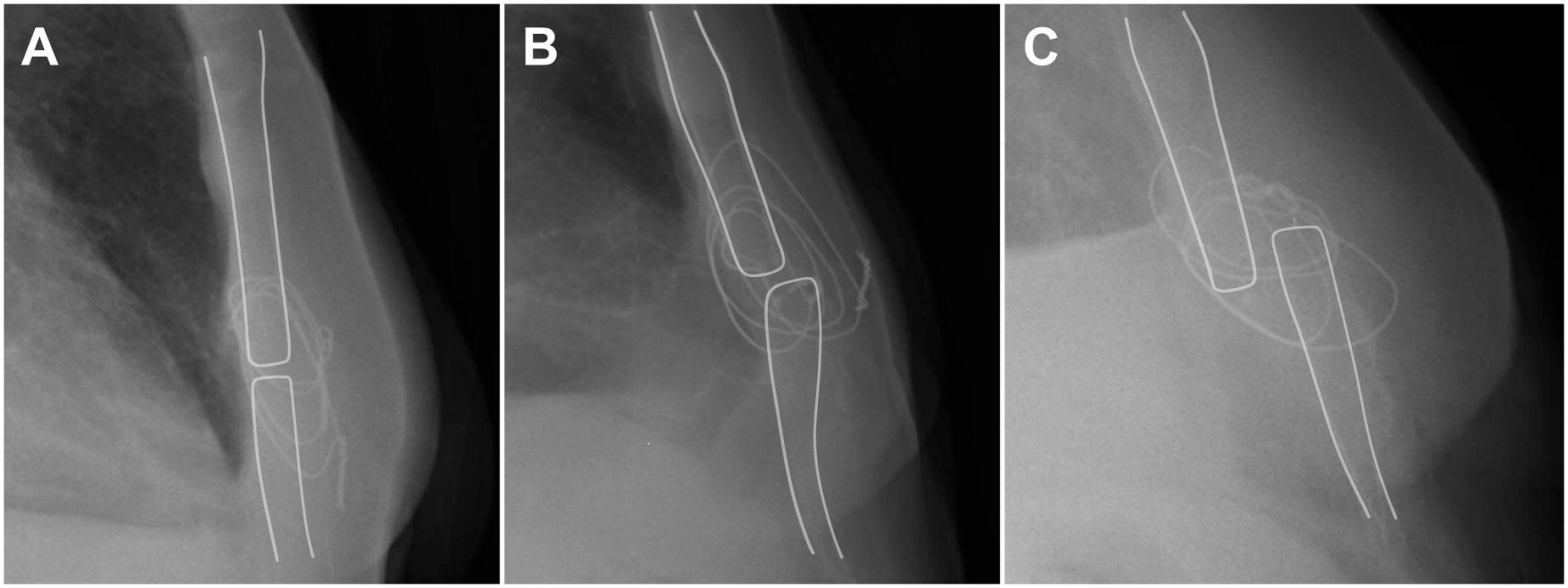
Lateral chest radiographs of three patients demonstrating different degrees of sternal alignment, according to the classification of Koster *et al*.^9^ (A) Normal alignment; (B) override; (C) separation. The sternal borders are highlighted with white lines to aid visualization.

Sternal alignment was assessed on postoperative lateral CXR, according to the classification proposed by Koster *et al*.^9^ Sternal override is a partial displacement but maintained overlap of the 2 sternal parts, whereas sternal separation is the complete displacement of the 2 sternal parts, which usually entails the sliding of the 2 segments one over the other in the sagittal axis (**Figure 2**).

In patients with multiple CXR available, we normally examined all those performed until the second postoperative month. A variation in the observed sternal alignment score since the first lateral CXR was uncommon. In that case, we considered the worst alignment score. If patients underwent a chest CT scan during the postoperative course, this was also reviewed for confirmation of the score. The images were independently reviewed by 2 specialists in thoracic surgery (C.A.S and G.R.) with at least 5 years of experience. In cases of disagreement, consensus was reached by discussion or by involving a third surgeon (M.M), with more than 10 years of experience.

Morphine milligram equivalents (MMEs) were calculated using standardized conversion factors. For tramadol, a conversion factor of 0.1 was used.^15^

### Statistical analysis

Continuous variables are presented as medians with interquartile ranges (IQRs), while categorical variables are reported as absolute numbers and percentages.

For the longitudinal analysis of pain, we collected NRS pain scores at postoperative days (POD) 7, 14, 21, 28, and at the first follow-up visit, which was scheduled at postoperative day 60. Each day, we took the average value among the 3 daily assessments of pain.

Clinical characteristics and outcomes between the patient groups were compared by Fisher’s exact test for categorical variables and Wilcoxon rank sum test for numerical variables. Univariable logistic regression analysis was performed to find risk factors for sternal separation. All clinically meaningful risk factors were selected for entry into a multivariable model. The final model was selected by stepwise elimination based on the Akaike information criterion (AIC). As a sensitivity analysis, a propensity score for sternal reapproximation technique (CWS vs other) was estimated using logistic regression, and inverse probability of treatment weighting (IPTW) was applied to weight patients in the multivariable (doubly robust) model evaluating sternal separation. Variables used for propensity score estimation included age, primary lung disease, preoperative ventilation, preoperative ECMO support, body mass index (BMI), lung allocation score (LAS), and preoperative steroid use. Covariate balance after weighting was assessed using standardized mean differences (SMD), and propensity score overlap between the groups was visually inspected and found to be adequate. *P* values were 2 tailed at the .05 level and unadjusted for multiplicity. All analyses were performed using the R software (R Foundation for Statistical Computing, Vienna, Austria). The authors did not use any generative AI tools in the preparation of this article.

## RESULTS

During the study period, 214 patients underwent BLTX at our centre. Of these, 164 cases were performed through a clamshell incision and had complete information on all variables collected, including at least one lateral CXR available for review.

The FRS group included 44 patients, the IWS group 10 patients, and the CWS group 110 patients. The majority of baseline clinical characteristics (summarized in **Table 1**), including the type of primary lung disease, LAS, BMI, prevalence of diabetes mellitus, and osteoporosis were comparable between the 3 groups. However, patients in the CWS group had a lower prevalence of ventilator and ECMO support prior to transplantation compared to other groups (**Table 1**). VA-ECMO for intraoperative support was used in the majority of cases (95.1%); while, postoperatively, patients in IWS had a higher rate of continuation of ECMO support (50%, compared to 25% and 12.7% in the FRS and CWS groups, respectively). In-hospital morbidity and mortality rates were comparable (53% and 7.9%, respectively).

**Table 1.** Baseline Characteristics of the Study Cohort

Characteristic	FRS, *n* = 44	IWS, *n* = 10	CWS, *n* = 110	*P*-value
Age (years)	56.5 (40-60)	50 (38.5-55.75)	54 (44.25-61)	.582
Primary lung disease				.354
Interstitial lung disease	31 (70.5%)	6 (60.0%)	65 (59.1%)	
Cystic fibrosis	9 (20.5%)	1 (10.0%)	17 (15.5%)	
COPD	4 (9.1%)	2 (20.0%)	20 (18.2%)	
CLAD	0 (0.0%)	0 (0.0%)	5 (4.5%)	
PPH	0 (0.0%)	1 (10.0%)	3 (2.7%)	
Lung allocation score (LAS)	37.98 (34.04-44.68)	34.87 (33.12-40.76)	35.23 (32.86-39.09)	.061
Diabetes mellitus	6 (13.6%)	3 (30.0%)	22 (20.0%)	.408
Cardiac disease	1 (2.3%)	1 (10.0%)	7 (6.4%)	.315
Osteoporosis	9 (23.7%)	3 (42.9%)	39 (43.8%)	.092
Body mass index (BMI, kg/m^2^)	25.20 (20.95-27.40)	26.10 (24.10-27.40)	23.90 (19.82-26.55)	.100
Preoperative steroids	24 (55.8%)	5 (62.5%)	39 (36.4%)	.053
Preoperative ECMO	3 (6.8%)	2 (20.0%)	2 (1.8%)	.017
Preoperative ventilation	3 (6.8%)	2 (20.0%)	3 (2.7%)	.043
Transplant type				.507
Bilateral LTX	44 (100.0%)	10 (100.0%)	105 (95.5%)	
Bilateral re-LTX	0 (0.0%)	0 (0.0%)	5 (4.5%)	
Transplant setting				.137
Election	40 (90.9%)	8 (80.0%)	104 (94.5%)	
Emergency	4 (9.1%)	2 (20.0%)	6 (5.5%)	
Intraoperative ECMO	40 (90.9%)	10 (100.0%)	106 (96.4%)	.345
Prednisone dose (mg/kg/day)	0.44 (0.38-0.49)	0.47 (0.44-0.48)	0.47 (0.43-0.53)	.103
Postop. complications	24 (54.5%)	4 (40.0%)	61 (55.5%)	.695
Sternal approximation				<.001
Normal	7 (15.9%)	3 (30.0%)	47 (42.7%)	
Override	19 (43.2%)	4 (40.0%)	53 (48.2%)	
Separation	18 (40.9%)	3 (30.0%)	10 (9.1%)	
Sternal revision surgery	0 (0.0%)	1 (10.0%)	1 (0.9%)	.191
Re-thoracotomy	9 (20.5%)	2 (20.0%)	16 (14.5%)	.624
Surgical site complications	7 (15.9%)	2 (20.0%)	16 (14.7%)	.758
Postop ventilation time (h)	60 (42-168)	72 (24-120)	48 (24-120)	.123
Postoperative ECMO	11 (25.0%)	5 (50.0%)	14 (12.7%)	.008
In-hospital death	4 (9.1%)	2 (20.0%)	7 (6.4%)	.228

Numerical data are reported as median (IQR).

Abbreviations: CLAD, chronic lung allograft dysfunction; COPD, chronic obstructive pulmonary disease; ECMO, extracorporeal membrane oxygenation; CWS, crossed wired sutures; FRS, figure-of-8 resorbable suture; IWS, interrupted wired sutures; LTX, lung transplantation; PPH, primary pulmonary hypertension.

The sternal alignment score was significantly different between the groups, with a lower proportion of patients in the CWS group having sternal separation (9.1%, compared to 30% and 40.9% in the IWS and FRS groups, respectively, *P* < .001). Overall, only 2 patients required revision surgery due to clinical signs of sternal dehiscence (1 in the CWS and 1 in the IWS groups). In both cases, sternal revision surgery preceded the date of the first available lateral CXR. Furthermore, sternal separation was not associated with a statistically significant increase in duration of mechanical ventilation, late complications, or in-hospital mortality (**Table S1**).

**Figure 3.**
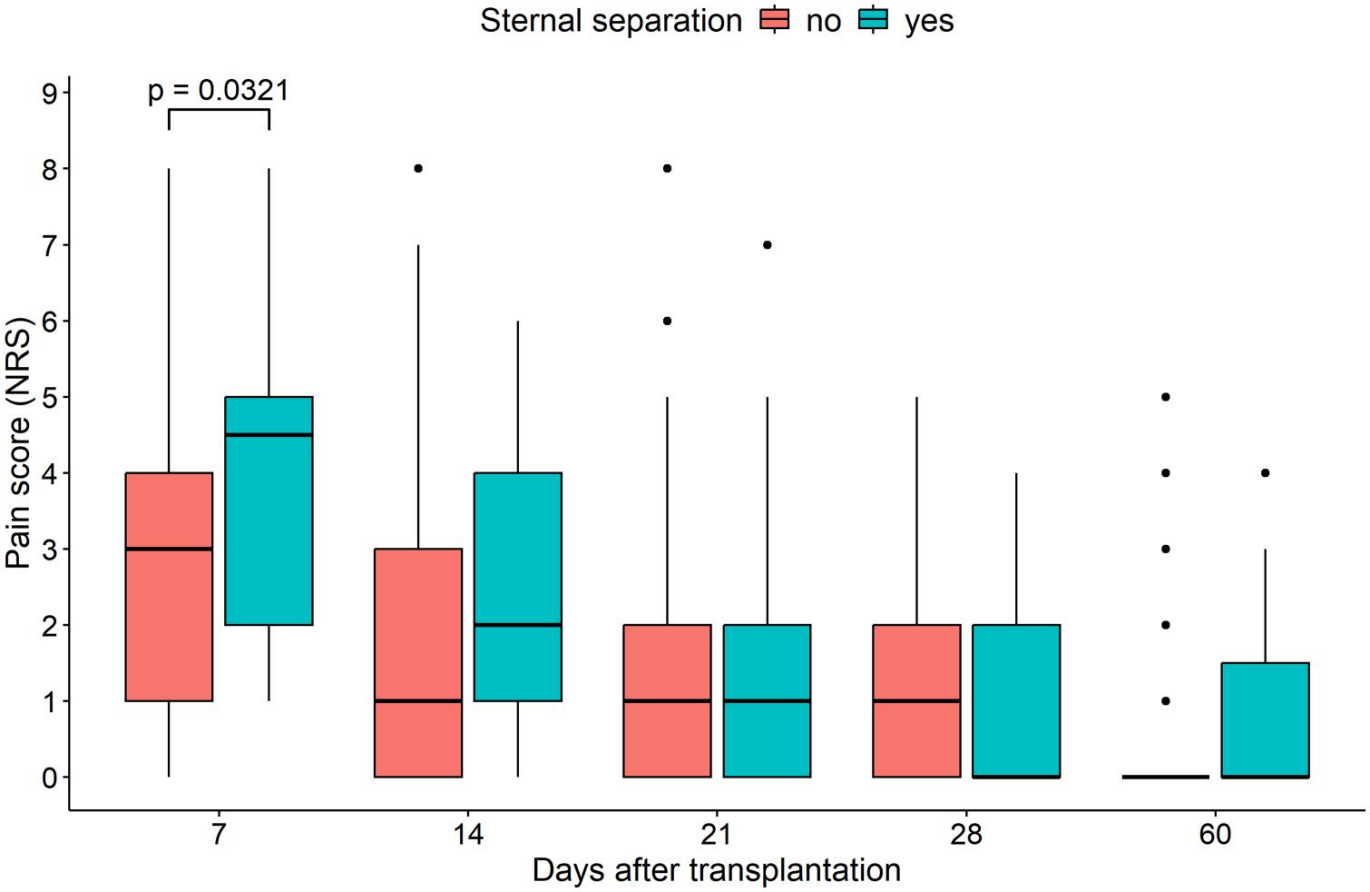
Paired box plots comparing the Numeric Rating Scale (NRS) pain scores at different time points between patients with sternal separation and those with either normal alignment or sternal override.

Pain scores were compared between patients with sternal separation and those with either sternal override or normal alignment at postoperative days 7, 14, 21, 28, and at the first follow-up visit (**Table 2** and **Figure 3**). Patients with sternal separation had higher pain scores on POD7, (*P* = .032), and a trend towards higher pain scores was observed on POD4 (*P* = .056), while pain scores at other time points and cumulative MME were comparable. When comparing pain scores according to the sternal closure technique (CWS vs other) no statistically significant difference was observed at any time point (**Figure S1**).

**Table 2.** Postoperative Pain Scores and Opioid Consumption According to Sternal Separation on Chest X-Ray

	Sternal separation		
Outcome	No (*n* = 134)	Yes (*n* = 31)	*P*-value
NRS pain score			
POD 7	3 (1-4)	4.5 (2-5)	.032
POD 14	1 (0-3)	2 (1-4)	.056
POD 21	1 (0-2)	1 (0-2)	.769
POD 28	1 (0-2)	0 (0-2)	.509
POD 60	0 (0-0)	0 (0-2)	.448
Opioid consumption			
Cumulative MME (POD28)	40 (0-130)	35 (0-130)	.839

Abbreviations: NRS, numeric rating scale; POD, postoperative day; MME, morphine milligram equivalents.

Univariable logistic regression for the risk of sternal separation identified a higher BMI as risk factors (odds ratio [OR]: 1.13, 95% confidence interval [CI]: 1.03-1.25); while the CWS technique of sternal reapproximation, compared to the others, was associated with a reduced risk (OR: 0.16, 95% CI: 0.06-0.036). At multivariable analysis, both BMI and the technique of sternal reapproximation remained independent predictors of sternal separation (*P* < .05, **Table 3**). After IPTW adjustment for baseline clinical characteristics, good balance was observed (SMD < 0.1) for all covariates (**Figure S2**). The weighted regression analysis confirmed the independent effect of BMI (OR: 1.16, 95% CI: 1.02-1.31) and the technique of sternal reapproximation (CWS vs other, OR: 0.15, 95% CI: 0.06-0.37) on the risk of sternal separation (*P* < .05, **Table 3**).

**Table 3.** Univariable, Multivariable, and Doubly Robust (IPTW-Weighted Multivariable) Logistic Regression for the Risk of Sternal Separation

	Univariable analysis		Multivariable analysis		IPTW-weighted multivariable analysis	
Characteristic	OR (95% CI)	*P*-value	OR (95% CI)	*P*-value	OR (95% CI)	*P*-value
Age	1.01 (0.98-1.04)	0.555				
Primary lung disease						
Other	Ref.					
Interstitial lung disease	1.15 (0.52-2.67)	0.732				
Lung allocation score (LAS)	1.01 (0.98-1.04)	0.526				
Body mass index (BMI, kg/m^2^)	1.13 (1.03-1.25)	0.015	1.13 (1.02-1.27)	0.029	1.16 (1.02-1.31)	.020
Diabetes mellitus	1.00 (0.34-2.55)	0.995				
Osteoporosis	1.04 (0.42-2.47)	0.936				
Prior steroid treatment	1.22 (0.54-2.70)	0.632				
Transplant setting						
Election	Ref.					
Emergency	0.77 (0.12-3.08)	0.744				
Preoperative ECMO	1.47 (0.21-6.77)	0.647				
Preoperative ventilation	0.60 (0.03-3.58)	0.644				
Technique of sternal closure						
Other	Ref.		Ref.		Ref.	
Crossed wired suture	0.16 (0.06-0.36)	<0.001	0.14 (0.06-0.34)	<0.001	0.15 (0.06-0.37) <.001	
Prednisone dose (mg/kg/day)	0.65 (0.03-14.4)	0.778				
Intraoperative ECMO	0.36 (0.08-1.84)	0.181				
Re-thoracotomy	0.98 (0.31-2.66)	0.969				
Postoperative ECMO	0.59 (0.16-1.66)	0.356	0.34 (0.08-1.09)	0.090	0.34 (0.09-1.23)	.094

Abbreviations: IPTW, inverse probability of treatment weighting, CI, confidence interval; ECMO, extracorporeal membrane oxygenation; OR, odds ratio.

## DISCUSSION

The most common surgical approaches to BLTX include clamshell thoracotomy, bilateral anterior thoracotomy, and median sternotomy.^16^ Each of them has its advantages and drawbacks, and the choice also depends on the intended policy regarding intraoperative extracorporeal life support. Because the clamshell thoracotomy provides optimal surgical exposure and facilitates central cannulation, it was selected as the standard approach at our centre. This choice reflects both our role as a teaching hospital and the routine use of central cannulation within a VA-ECMO-based strategy. Nonetheless, several authors have demonstrated the feasibility of performing BLTX with intraoperative extracorporeal life support even through less invasive approaches, including bilateral small thoracotomies and robot-assisted lung transplantation.^17^

One of the main concerns related to the clamshell incision is the risk of sternal complications, which, according to earlier reports, was as high as 34%-45%,^3^^,^^4^^,^^18^ while in more recent studies it ranged from 8% to 17%.^9^^,^^19^ An important issue, however, is represented by the different definitions of sternal complication. In fact, a misalignment of the 2 sternal ends may not always require a reoperation, but it could cause sternal instability or chronic pain.^5^

Koster *et al* proposed a novel radiological classification of the alignment of the 2 sternal ends that is easy to assess on lateral CXR and that, for consistency, we adopted in the present study (**Figure 2**).^9^ We found that sternal alignment could be easily assessed retrospectively by thoracic surgeons using this classification, with only rare instances of interobserver disagreement.

In the study by Koster *et al*, 56 on 129 patients (43%) had either a sternal override (30%) or sternal separation (14%), and only patients who had sternal separation required a reoperation (10 on 18 patients). In our study, the proportion of patients with override or separation was slightly higher (46.3% and 18.9%, respectively), but only 2 patients were reoperated on for clinical signs of sternal dehiscence. The reasons for this discrepancy are uncertain, and deserve to be further investigated. A possible explanation is that clinicians may have different perceptions of the negative effect of a misalignment of the sternal ends on the postoperative course, and, therefore, the threshold for prompting a reoperation may also be different.

To the best of our knowledge, this study is the first to demonstrate that a severe misalignment of the sternal ends (eg, sternal separation) is associated with significantly higher pain scores at POD7 from BLTX, and a trend towards higher pain scores at POD14 (*P* = .056, **Figure 3**). This finding, one hand supports the clinical notion that a poor alignment of the sternum is an undesirable finding, even when it does not result in a reoperation; while, on the other hand, it highlights the utility of this radiological classification to describe a phenomenon (eg, sternal alignment) that is of clinical relevance in the early postoperative weeks following BLTX. When pain scores were compared according to the sternal closure technique (CWS vs other), no significant difference was observed (**Figure S1**). This finding supports the hypothesis that pain is caused directly by the occurrence of sternal separation; which could not be entirely prevented by the CWS closure technique.

Similar to the present study, Koster *et al* aimed to compare different techniques of sternal reapproximation for their efficacy on postoperative sternal alignment. The authors used an uncrossed wired suture (which corresponds to our IWS technique) in 79 patients and a sternal or a parasternally crossed wired suture in 50 patients, experiencing significantly lower override and separation rates in the crossed wired suture group.^9^

The technique that we used on the CWS group is similar to the parasternally crossed wired technique of Koster *et al*, with the difference that the wire ends lie over the body of the sternum rather than across the intercostal space (**Figure 1C**). We feel that, in this manner tightening and twisting the wire ends results in a precise and stable reapproximation of the sternal stumps by channelling compressive forces centrally, whereas tightening the wire ends over the intercostal space may increase the risk of rib fracturing.

Overall, we found, on a larger cohort than prior studies, that the CWS technique was more effective in preventing sternal separation (9.1% compared to 40.9% and 30.0% in the FRS and IWS groups, respectively). Moreover, multivariable analysis confirmed that the reapproximation technique is independently associated with sternal separation, and that a higher BMI is another important risk factor for such event, with an increased risk of 13% for each one-point increase in BMI (*P* = .029, **Table 2**). A similar effect of higher BMI on the risk of sternal dehiscence was observed also in the cardiac surgical literature after median sternotomy.^20^^,^^21^

Rigid plate fixation is another option for sternal reapproximation after BLTX, which has been reported to provide promising outcomes in small retrospective series.^6^^,^^7^^,^^22^ Although comparative studies in the field of lung transplantation are lacking, by borrowing the evidence in the field of cardiac surgery, it may be presumed that rigid plate fixation leads to better outcomes in terms of sternal stability and pain than metal wires.^23^ On the other hand, sternal fixation with rigid plates is associated with higher costs, longer operative times, and the need for dedicated equipment. These factors raise concerns regarding its long-term feasibility as a routine method of sternal reapproximation in BLTX. Nevertheless, the biomechanical superiority of rigid plates may still be preferable in high-risk cases (high BMI or redo cases) where enhanced stability could justify the additional cost and operative time.^23–25^

Besides its retrospective nature, this study has several limitations. First, the sample sizes across different sternal closure techniques were markedly unbalanced; therefore, patients undergoing IWS and FRS were grouped in the regression analysis. While this approach allows comparison between CWS and the other techniques, it precludes a separate assessment of the individual impact of IWS and FRS on the risk of sternal separation. Second, based on the observed trend in pain scores (**Figure 3**), the effect of sternal misalignment on pain may be even more pronounced in the early postoperative period (48-72 hours). However, pain assessments at these time points were unavailable for most patients due to ICU stay, intubation, or sedation, potentially leading to an underestimation of the clinical relevance of sternal misalignment. Third, information on wound complications, sternal stability or pain beyond 60 days were not available for assessment. Although bone callus formation is normally expected to occur within this time frame, the absence of later follow-up may still result in the loss of clinically meaningful information, highlighting an area for further investigation. Fourth, a change in institutional policy regarding intraoperative VA-ECMO was introduced during the study period; therefore, the possibility of an era bias cannot be completely excluded. However, the proportion of patients receiving intraoperative ECMO was comparable among the 3 groups (above 90%). Moreover, the study period was relatively short (7 years), which reduces the likelihood of major contemporaneous changes in patient management.

## CONCLUSION

To conclude, this is the largest study to date that compares different methods of sternal reapproximation following clamshell thoracotomy for BLTX. By using a pre-existing classification of the sternal alignment based on lateral CXR, we found that the CWS technique leads to better alignment than the FRS and IWS techniques. The effect of the reapproximation technique was independent from other variables, such as the BMI, which also increases the risk of sternal separation. Contrarily to a previous study, we did not find an association between sternal misalignment and the need for sternal revision surgery. The reasons for this discrepancy are unknown and deserve further investigation. On the other hand, this is the first study in the field of lung transplantation to demonstrate an association between a severe sternal misalignment and higher pain scores during the first 2 postoperative weeks. Based on these findings, we suggest the CWS technique as the routine method for sternal reapproximation following clamshell thoracotomy for BLTX, although rigid plate fixation may be considered in high-risk cases.

## Supplementary Material

ivag079_Supplementary_Data

## Data Availability

The data that support the findings of this study are available from the corresponding author upon reasonable request.
