# Supplementary material for: Sternal Closure After Clamshell Thoracotomy for Bilateral Lung Transplantation: Comparison Between Different Techniques
Source: Interdiscip Cardiovasc Thorac Surg. 2026 Mar 18;41(3):ivag079. doi: 10.1093/icvts/ivag079 (PMC13032823; doi:10.1093/icvts/ivag079)

**Supplementary Table 1**: Baseline characteristics and outcomes of the study cohort according to the sternal alignment score

|  | Sternal alignment score | | | |
| --- | --- | --- | --- | --- |
| Characteristic | **Normal, n = 58** | **Override, n = 76** | **Separation, n = 31** | **p-value** |
| Age | 50 (39.25 - 61) | 57 (44.75 - 62.25) | 56 (46.5 - 60) | 0.116 |
| Primary lung disease |  |  |  | 0.283 |
| CLAD | 2 (3.4%) | 3 (3.9%) | 0 (0.0%) |  |
| COPD | 9 (15.5%) | 11 (14.5%) | 6 (19.4%) |  |
| Cystic fibrosis | 11 (19.0%) | 12 (15.8%) | 5 (16.1%) |  |
| Interstitial lung disease | 32 (55.2%) | 50 (65.8%) | 20 (64.5%) |  |
| PPH | 4 (6.9%) | 0 (0.0%) | 0 (0.0%) |  |
| Lung Allocation Score (LAS) | 34.22 (32.78 - 38.47) | 36.43 (33.93 - 42.43) | 35.06 (32.96 - 44.01) | 0.140 |
| Diabetes mellitus | 16 (27.6%) | 10 (13.2%) | 6 (19.4%) | 0.112 |
| Cardiac disease | 2 (3.4%) | 5 (6.6%) | 2 (6.5%) | 0.705 |
| Osteoporosis | 16 (35.6%) | 25 (39.1%) | 10 (38.5%) | 0.930 |
| Body Mass Index (BMI) | 22.60 (19.11, 26.98) | 24.15 (20.48, 26.16) | 26.20 (24.20, 28.50) | **0.017** |
| Prior steroid treatment | 23 (41.8%) | 31 (41.9%) | 14 (46.7%) | 0.891 |
| Transplant type |  |  |  | 0.543 |
| Bilateral LTX | 56 (96.6%) | 73 (96.1%) | 31 (100.0%) |  |
| Bilateral re-LTX | 2 (3.4%) | 3 (3.9%) | 0 (0.0%) |  |
| Transplant setting |  |  |  | 0.937 |
| Election | 53 (91.4%) | 70 (92.1%) | 29 (93.5%) |  |
| Emergency | 5 (8.6%) | 6 (7.9%) | 2 (6.5%) |  |
| Sternal closure |  |  |  | **<0.001** |
| FRS | 7 (12.3%) | 19 (25.0%) | 18 (58.1%) |  |
| CWS | 47 (82.5%) | 53 (69.7%) | 10 (32.3%) |  |
| IWS | 3 (5.3%) | 4 (5.3%) | 3 (9.7%) |  |
| Intraop. ECMO | 56 (96.6%) | 73 (96.1%) | 28 (90.3%) | 0.378 |
| Sternal revision surgery | 0 (0%) | 1 (1.3%) | 1 (3.2%) | 0.799 |
| Re-thoracotomy | 8 (13.8%) | 14 (18.4%) | 5 (16.1%) | 0.772 |
| Surgical site complication | 5 (8.8%) | 16 (21.1%) | 4 (12.9%) | 0.138 |
| Postop ventilation time (hours) | 48 (24- 144) | 48.00 (24 - 139) | 48.00 (24 - 72) | 0.610 |
| Postop ECMO | 13 (22.4%) | 14 (18.4%) | 4 (12.9%) | 0.546 |
| Late complications | 12 (21.1%) | 20 (26.7%) | 7 (22.6%) | 0.741 |
| In-hospital death | 8 (13.8%) | 5 (6.6%) | 0 (0.0%) | 0.060 |
| CLAD: Chronic lung allograft dysfunction; COPD: Chronic obstructive pulmonary disease; PPH: Primary pulmonary hypertension; LTX: Lung transplantation; FRS: Figure-of-eight resorbable sutures; CWS: Crossed wired sutures; IWS: Interrupted wired sutures; ECMO: Extracorporeal membrane oxygenation. | | | | |

**Supplementary Figure 1**: Paired boxplots comparing the numeric rating scale (NRS) pain scores at different time points according to the technique of sternal closure (CWS: crossed wired suture)


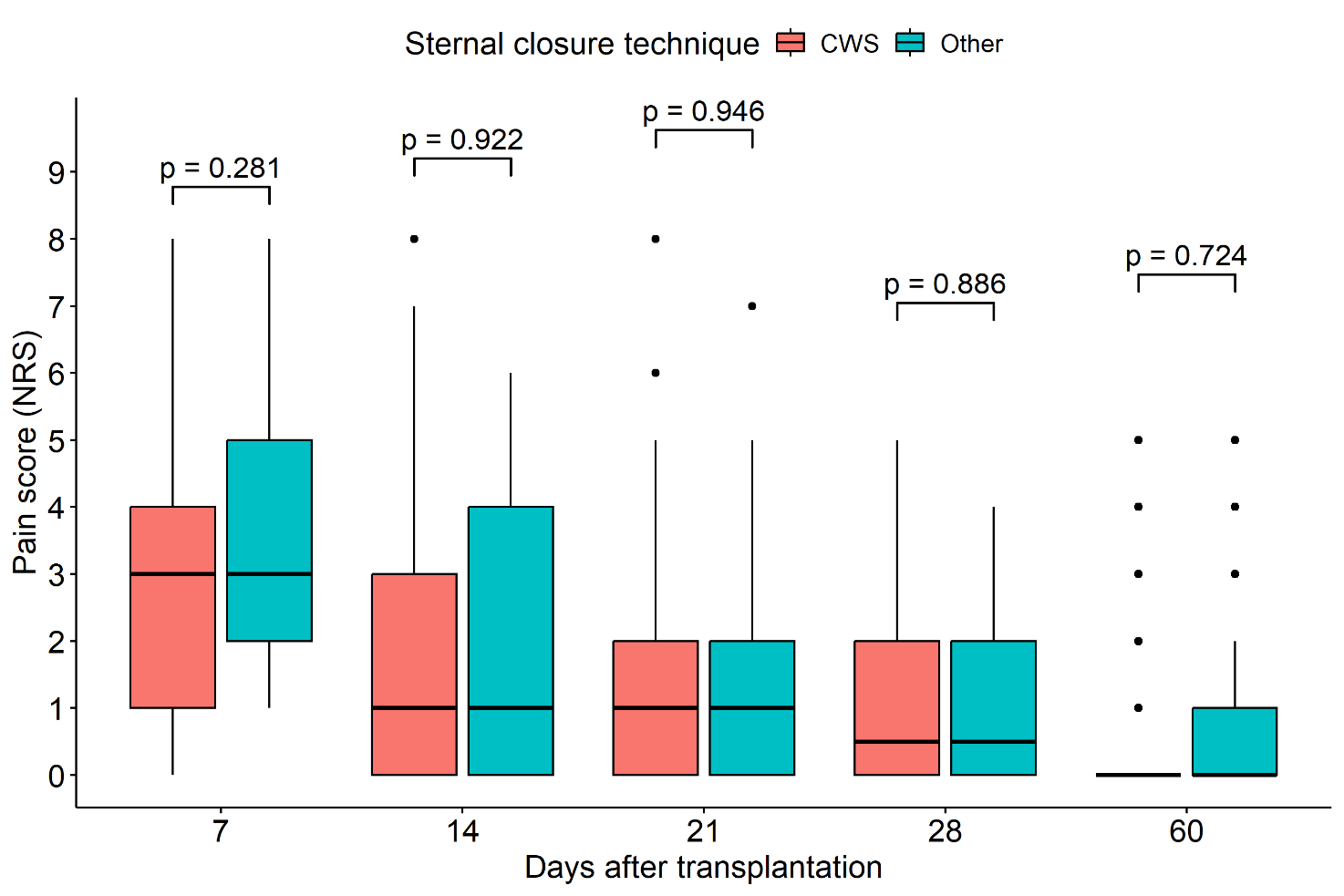


**Supplementary Figure 2**: Covariate balance before and after propensity score weighting


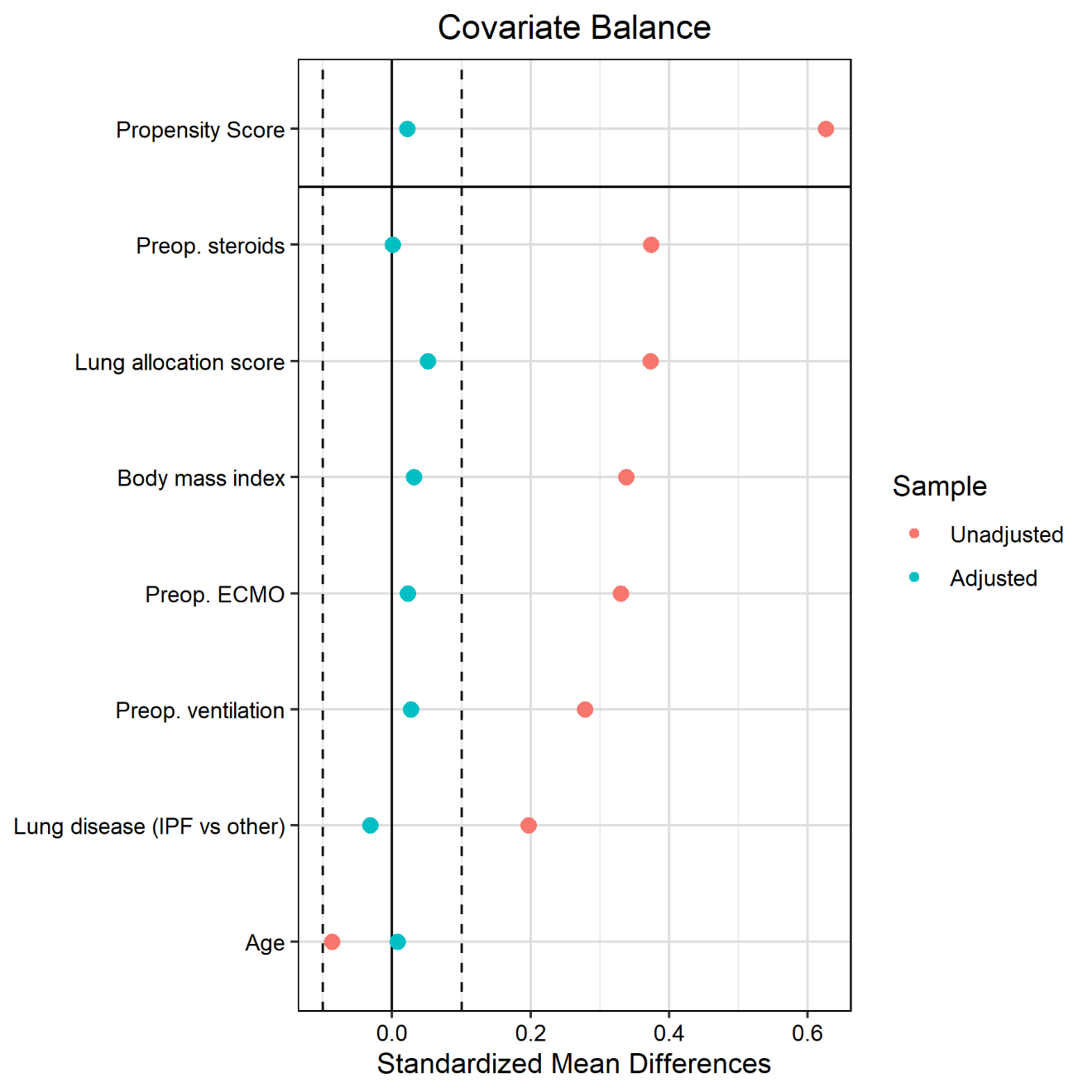

Supplement: ivag079_Supplementary_Data [file ivag079_supplementary_data.docx]
